# Academic Detailing is a Preferred Knowledge Update Tool Among Norwegian Pharmacists to Improve Antibiotic Counseling: Results From a Quantitative Study Employing the Provider Satisfaction With Academic Detailing (PSAD) and the Detailer Assessment of Visit Effectiveness (DAVE) Tools

**DOI:** 10.1177/00469580241273228

**Published:** 2024-09-04

**Authors:** Yngvild Kristine Rochette Bergsholm, Harald Christian Langaas, Tonje Krogstad, Lene Berge Holm

**Affiliations:** 1 Department of Life Sciences and health, Faculty of Health Sciences, Oslo Metropolitan University, Oslo, Norway; 2 Trondheim Hospital Pharmacy, Central Norway Pharmaceutical Trust, Trondheim, Norway; 3 Health Services Research Unit, Akershus University Hospital, Lørenskog, Norway

**Keywords:** antimicrobial resistance, pharmacists, patient-centered care, academic detailing, AD, adherence, Provider Satisfaction with Academic Detailing (PSAD), Detailer Assessment of Visit Effectiveness (DAVE)

## Abstract

Excessive and incorrect use of antibiotics contributes to the rise of antimicrobial resistance (AMR). Given that pharmacists act as final checkpoint before antibiotics is handled over to patients, they play a crucial role in promoting proper antibiotic use and ensuring treatment adherence. However, there is often a gap between the patients’ needs and perceptions, and what the pharmacists provide. Improving pharmacists’ training is essential for enhancing patient-centered care. The aim of this research was to evaluate the suitability of academic detailing (AD) for improving Norwegian pharmacists’ knowledge and practice on adherence promoting counseling of antibiotic patients. Key insights from prior qualitative research regarding community pharmacists’ position in promoting optimized antibiotic use were incorporated in a tailored AD program. The AD’s suitability was evaluated using the validated “Provider Satisfaction with Academic Detailing” (PSAD) and “Detailer Assessment of Visit Effectiveness” (DAVE) instruments. Additionally, participants preferred knowledge updates method were assessed. Eighty-one of 86 visits completed PSAD (94% response rate). Satisfaction summary score for PSAD was 40.03 (of maximum 45) and scale summary score for DAVE 12.45 (of maximum 15). One-sample *t*-test (P < .001) indicated preference for AD over other knowledge update methods. This study confirmed that AD is a successful knowledge updating tool for improving adherence promoting counseling among Norwegian pharmacists. Future research should align practice change intentions with actions post-AD and evaluate patient impact.

**What do we already know about this topic?**
We know that Academic Detailing has proven to be effective as a tool for enhancing the service of general practitioners.
**How does your research contribute to the field?**
Our research contributes to the field by evaluating the impact of utilizing Academic Detailing toward community pharmacists.
**What are your research’s implications toward theory, practice, or policy?**
Our research has implications for pharmaceutical practice as it demonstrates to be an effective tool for improving pharmacists’ patient centered counseling of antibiotic patients.

## Introduction

Globally, there is significant concern surrounding the inappropriate use of antibiotics and its association with increased antimicrobial resistance (AMR).^1-5^ In Norway, approximately 85% of prescriptions for antibiotics are issued in primary care by general practitioners (GPs). Consequently, several antibiotic stewardship programs (AMS) have been directed at GPs, with the aim of promoting evidence-based prescribing.^3,6,7^ A successful implementation of AMS necessitates a collaborative approach involving a multidisciplinary healthcare team, which include pharmacists.^6,8,9^ Although pharmacists do not have a role in the prescribing of antibiotics in Norway, they have a crucial position as the final checkpoint when patients pick up their medications at the pharmacy. The pharmacist can then provide the final counseling before patients administer their prescribed antibiotics by themselves at home.^10,11^ In this capacity, the pharmacists can play a pivotal role in promoting proper antibiotic use and ensuring adherence to the prescription.^10^ Furthermore, improved patient care has been observed when GPs and pharmacists collaborate.^10^

In Norway, community pharmacies are primarily owned by 3 large pharmacy chains, with only a few privately own pharmacies.^12^ Many urban pharmacies, with their extended hours, provide easy access to pharmacists, making their competences highly accessible.^10^ The current practice of Norwegian community pharmacists involves distributing medications, providing information, and offering patient-centered care to ensure safe and medically appropriate use of medications.^13^ However, Svensberg et al^14^ have demonstrated that Norwegian community pharmacists’ vision of patient-centered care is hindered by their current practices and agent relationships. The traditional product-focused culture limits their ability to fully engage in their roles and advance patient care.

Prior research has indicated that patients demonstrate a receptivity to acquiring information by pharmacists at pharmacies.^15-21^ Patients generally regard pharmacists as knowledgeable and valuable, fostering a less hierarchical dialog using easier everyday languages in contrast to GPs. This resulting in information that is more easily comprehensible.^10^ Despite this, the pharmacists’ potential to contribute to an improved use of medication is underutilized.^22^ A discrepancy has been identified between the type of interaction and information that patients sought from pharmacists and what was actually provided.^14,23^ To improve health outcomes, pharmacists should undergo training to enhance their patient-centered care skills in patient interactions.^23-26^

Academic detailing (AD) is a knowledge update method originally aimed at enhancing prescription practices. It involves delivering a concise overview of updated evidence pertaining to a specific topic during a one-on-one dialog with the prescriber.^27,28^ The interaction and dialog between the visitor, a trained healthcare professional - often referred to as an academic detailer, and the recipient of information is considered essential. The academic detailer is trained to recognize the unique needs of each recipient and tailor the information accordingly. The AD intervention tool has effectively promoted changes in prescribing behaviors across a broad spectrum of medical treatments^28-30^ with a primary focus at GPs on drug therapy to enhance prescription adherence within specific clinical areas.^29,31-36^ Furthermore, such individualized visits are more suitable in promoting practice change than group meetings.^28,33,37,38^

The “Provider Satisfaction with Academic Detailing” instrument (PSAD) and the “Detailer Assessment of Visit Effectiveness” instrument (DAVE) are designed for evaluating the effectiveness of AD-programs.^39,40^ The findings from PSAD questionnaires in Smart et al’s^33^ indicated a substantial level of satisfaction with AD visits.

In Norway previous studies have demonstrated that AD has effectively influenced the GP’s prescription practices.^28-30^ Therefore, it is intriguing to investigate whether AD particularly focusing on antibiotics, can also function as a tool for knowledge updates for pharmacists. To our knowledge, there has not been any academic detailing (AD) campaign specifically tailored and utilized to update the knowledge and practice of Norwegian pharmacists to date. The aim of this study is to evaluate the suitability of AD as a tool for improving the knowledge and practice of Norwegian pharmacists in relation to promoting better adherence to antibiotics.

## Methods

This study employs a quantitative evaluation approach using validated instruments to assess the effectiveness of an Academic Detailing program tailored for Norwegian pharmacists.

### Recruitment

Pharmacies in Oslo area, Norway, were selected for participation through convenience sampling with open invitations via telephone calls and emails. The only inclusion criterion for the recruited pharmacies was to be a regular community pharmacy in Oslo and the surrounding area. The inclusion criterion for the pharmacists participating in the AD visits was to be employed in one of the recruited pharmacies and to either hold a master’s or bachelor’s degree in pharmacy or to be a licensed pharmacy student. Initially, the academic detailer contacted the pharmacy managers to provide verbal information about the project’s purpose and the involvement required. Subsequently, a written summary was sent via email. Some pharmacies chose to participate immediately during the phone call, while others preferred to consult with colleagues before deciding. Additionally, certain pharmacy managers declined either during the phone call or later through email.

### Intervention

The AD visits took place in March 2023, each consisted of a single one-on-one meeting between the pharmacist and the academic detailer. One academic detailer conducted all 86 visits. The detailer holds a master’s degree in pharmacy and has substantial work experience on antibiotic related aspects. This research is a part of the PhD education. The detailer was trained in AD through a course provided by the Regional Medicines Information and Pharmacovigilance Centre (RELIS)^41^ to ensure a consistent presentation of the topic while also tailoring the interaction based on individual needs, determined by how the recipients responded to questions. Visits were scheduled for 20 min for each pharmacist.

According to the AD method, a four-page brochure was prepared prior the visits to support the oral message. A team of researchers collaborated to distil the most crucial insights from previous qualitative research on the topic of adherence to antibiotics and relational communication between patients, pharmacists and physicians.^10,42^ A model of a typical AD visit, and the brochure can be seen in Supplemental Material. Three key messages were identified and presented on the front of the brochure. The brochure’s remaining content presents evidence, supplementary background information, and reference citations to effectively convey the essential aspects of the topic for a concise knowledge update.

### Data Collection

Subsequent to each visit, the detailer electronically assessed the experience using the DAVE instrument. DAVE is a five-item questionnaire, developed and validated by researchers at the University of Illinois,^39^ wherein the aspects of the AD visit are evaluated by the detailer based on a 5-point Likert scale. The response options were as follows: “Not at all” = 1, “Slightly” = 2, “Moderately” = 3, “Very” = 4, and “Extremely” = 5. A detailer scale summary score for items 1 to 3 (usefulness, relevance, and acceptability) is calculated, yielding a score range from 3 to 15. Items 4 (feasibility) and 5 (communication) are reported separately, each with a range between 1 and 5.

After the AD visit, pharmacists were provided with a QR code for immediate access to and completion of the PSAD questionnaire. Also this questionnaire is developed and validated by the same researchers at the University of Illinois as that of the DAVE instrument.^39,40^ The responders’ answers were anonymous. The PSAD is a ten-item measure created to evaluate receivers’ satisfaction with the AD visits.^40^ The items were presented in English and covered: (1) knowledge, (2) effectiveness of communication, (3) effectiveness of AD, (4) usefulness, (5) willingness to repeat experience, (6) acceptability/relevance, (7) acceptability/importance, (8) feasibility, (9) consistency, and (10) willingness to change (WTC). These ten items were also scored on the same 5-point Likert scale as described for DAVE. A PSAD satisfaction summary score was generated by summarizing scores for item 1-9 which gives a range between 9 and 45. Item 10 (WTC Score) was reported separately with a range between 1 and 5.

Furthermore, participants were asked about their gender, educational background, and years of experience as community pharmacists. Participants were also asked about their preferred method for receiving knowledge updates with this question: Consider the method of knowledge updating you have just experienced and compare it with other methods you normally use to update your knowledge in your daily work. Indicate on a scale from 1 to 10 which method you prefer, where 1 indicates that you most prefer Academic Detailing visits, and 10 indicates that you most prefer other methods that you usually use. Additionally, participants were invited to provide comments in an open-text section as a complement to this inquiry or the other questions in the survey.

### Statistical Analysis

Sample size calculations were conducted before data collection. Although there was no intention of statistically comparing DAVE and PSAD results, power calculation was performed as if these results would be compared to those of Smart et al.^33,39,40^ In a significant study using PSAD, a satisfaction summary score of 41.3 with an SD of 4.70 was reported for in-person opioid prescriber visits.^33^ Based on this, assuming a clinically relevant score difference of 2.5, an alpha of 0.05, and power of 0.8, the required sample size is 55 per group.

Participants rated their preferred method of receiving knowledge updates on a scale from 1 to 10, with 5.5 as the neutral midpoint. A symmetrical distribution of responses around this midpoint would indicate no statistically significant preference for either method of knowledge update. The mean and standard deviation (SD) were calculated and compared to the midpoint value using a one-sample *t*-test. Sample size calculations for this test, using a population mean of 5.5, a hypothesized SD of 3.0, a clinically relevant score difference of 1.0, an alpha of 0.05, and power of 0.8, indicated a required sample size of 71.

Data analysis was conducted using Excel Toolpak version 16.0.12527.20612.

The underlying materials from this research can be made available by contacting the corresponding author.

## Results

The characteristics of the respondents are summarized in Table 1. The mean duration of the AD-visits was 31.24 min (SD 4.18).

**Table 1.** Description of Participants.

Years of practice	0-5: 42 6-10: 17 11-15: 12 16-20: 5 21-25: 2 26-30: 2 31-35: 0 35-40: 1
Education level	Master in pharmacy: 39 Bachelor in pharmacy: 33 Student with license: 9
Gender	Male: 17 Female: 62 Not answered: 2
Visits in number	86
Non-responders	5

### PSAD and DAVE Questionnaires

A total of 86 academic visits were carried out, and 81 of these visits included the completion of the PSAD questionnaire, resulting in a response rate of 94%. Respondents were encouraged to complete the PSAD questionnaire after the visits, and the mean and SD for all 9 questions, in addition to the willingness to change their practice (WTC score) and satisfactory summary score, were computed (Table 2).

**Table 2.** Providers Satisfaction With Academic Detailing (PSAD).

Provider Satisfaction with Academic Detailing (PSAD)	N = 81 Mean (SD)
1. The detailer was knowledgeable	4.41 (0.54)
2. The detailer was an effective communicator	4.42 (0.57)
3. AD is an effective way to get an updated on important topic(s)	4.40 (0.70)
4. The printed material was useful	4.22 (0.61)
5. I would be receptive to future visits	4.36 (0.75)
6. The topic was relevant to my practice	4.68 (0.57)
7. This is an important topic	4.82 (0.45)
8. The key messages are feasible to implement in my practice	4.41 (0.63)
9. The key messages were consistent with my practice	4.31 (0.65)
**10. My practice is likely to change as a result of this visit (WTC score)**	**3.83 (0.86)**
**Satisfaction summary score**	**40.03 (3.85)**

The Satisfaction summary score (marked in bold) is the sum of scores 1 to 9. Score 10 equals the Willingness to change score (WTC), also marked in bold.

The results obtained from the PSAD questionnaires indicate a satisfactory score with a mean value of 40.03 (SD 3.85) out of a maximum possible score of 45. The mean WTC score was 3.83 (SD 0.86) out of a maximum of 5.

The academic detailer responded to the DAVE questionnaire after each visit, and the results from the DAVE scale are presented in Table 3. The mean detailer scale summary score in this study was 12.45 (SD 1.10) out of a maximum of 15.

**Table 3.** Academic Detailers Satisfaction With AD Visits, DAVE.

Detailer Assessment of Visit Effectiveness (DAVE)	N = 86 Mean (SD)
1. The visit was useful to the provider	4.10 (0.43)
2. The provider is willing to implement the key points	4.25 (0.49)
3. The provider is likely to change his/her/their practice as a result of this visit	4.09 (0.50)
**Scale summary score (item 1-3)**	**12.45 (1.10)**
4. It is feasible for the provider to implement the key points	4.11 (0.46)
5. The conversation went smoothly	4.57 (0.56)

### Preferred Method for Knowledge Update

**Figure 1.**
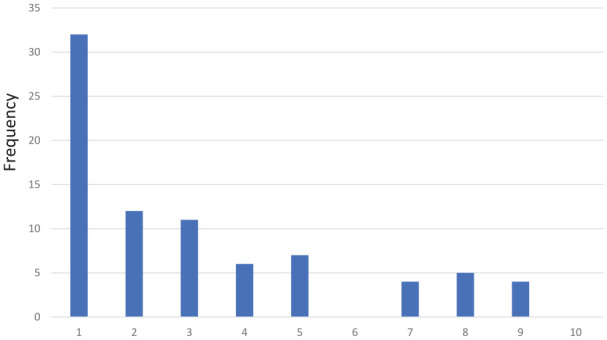
The frequency of responders preferring AD or other methods, based on the question “Consider the method of knowledge updating you have just experienced and compare it with other methods you normally use to update your knowledge in your daily work. Indicate on a scale from 1 to 10 which method you prefer, where 1 indicates that you most prefer Academic Detailing visits, and 10 indicates that you most prefer other methods that you usually use.”

The results of the question regarding preferred knowledge update methods are presented in Figure 1. The mean score for this question was 3.11 (SD 2.52). This score was compared with a reference midpoint value of 5.5 through a one-sample *t*-test, resulting in P < .001 indicating a significant preference for AD over other knowledge update methods with a significance level α of .05.

### Responses From Open Text Field

The pharmacists could provide justifications for their choice of the preferred method in an open-text section. The following supporting arguments for AD as a method of updating knowledge were provided: pharmacists found AD a suitable method for delivering evidence-based information, the customization of information to individual needs, and the direct interaction with detailers. One participant express:

“*Dialog face to face with detailer. Possibility to tailor information to the needs of each individual. Participants can ask questions directly. Individually adjusted for the participant*.”

Additionally, some pharmacists experienced it as saving time compared to participating in physical courses and online training.

*“Knowledge update visits are better than e-learning or courses because they are individually tailored to the participant’s need, have a shorter duration than courses, and offer a good opportunity to ask questions.”*

The pharmacists in this study emphasized the campaign’s theme as being useful. They highlighted that the AD material provided them with a tool to improve the triangular dialog between the patient, pharmacist, and GP ultimately having potential to maximize adherence to prescriptions. One pharmacist wrote:

“*Great initiative! We need everything we can to enhance the collaboration between pharmacists and physicians. It was incredibly important to clarify how pharmacists can utilize the leeway we have with patients*.”

These statements indicate that pharmacists gained valuable insights into the behavior and perspectives of both GPs and patients, which was part of the essential information provided both in the written material and orally during the AD visit, leading to a better understanding of how to be more flexible and effective during interactions with patients at the pharmacy. Some pharmacists expressed that the AD visit heightened their awareness of the significance of communication in enhancing patient adherence to antibiotic use.

Barriers to AD being the preferred method included the perception, especially among pharmacy managers, that the visits were time-consuming. Consequently, the pharmacies were deemed unsuitable for the updating approach. They faced difficulties in allocating time for the staff for this purpose, as illustrated by the following note:

*“Time constraints. Pharmacy operations are perceived as too busy to accommodate additional AD visits. It is challenging to allocate personnel resources for the visits.”*

In addition, concerns were raised that it will be time-consuming to tailor information material for various campaigns.

## Discussion

In a study by Smart et al^33^ AD was used for appropriate opioid prescribing. In the in-person visits they reported a mean satisfaction summary score at 41.30 (SD 4.70) as measured by the PSAD questionnaire. In our study, we observed a mean satisfaction summary score measure of 40.02 (SD 3.85). These findings suggest that our AD strategy with pharmacists in Oslo, Norway, demonstrates a similar level of feasibility and acceptability to that observed in Smart et al “s study. In a systematic review conducted by Kulbokas et al,^43^ 5 interventions^44-48^ further supported these findings and highlighted the effectiveness of AD as an intervention tool associated with high reported satisfaction, both in terms of feasibility and acceptability. Moreover, Smart et al^33^ reported a WTC score of 2.66 (SD 1.23) which is lower than our WTC score at 3.83 (SD 0.86). These findings may indicate that Norwegian pharmacists are inclined and motivated to adopt a patient-centered care approach, aligning with patients” preferences.^10^ This aligns well with the results from Svensberg et al^14^ who demonstrates that Norwegian community pharmacists’ vision of patient-centered care is hindered by their current practices. According to the Norwegian legislation^49^ pharmacists are required to continuously enhance their professional knowledge, which also might explain the high WTC score.

Previous studies have discovered that one of the primary roles expressed by pharmacists is bridging the communication gap between patients and general practitioners.^10,11^ This finding may explain why pharmacists willingly embraced these particular AD visits. One of the main focuses of these visits was on how pharmacists could leverage the opportunity when the patient is more receptive to information, which is often the case at the pharmacy rather than at the doctor’s office, as patients tend to feel more vulnerable at the doctor’s office.^10,42^ Pharmacists can bridge this communication gap by confirming, supplementing, informing, and correcting the information that patients have comprehended from their doctor’s visit. From a societal perspective, with the dramatic increase in AMR, it is crucial for pharmacists to be aware of patients’ empowerment dynamics, maintain their sense of control, and provide tailored information to ensure proper antibiotic use.

The pharmacists find AD campaigns to be a convenient means of staying updated on the most recent research. The one-on-one knowledge updating process is perceived as time-efficient by pharmacists, enabling them to maintain their focus effectively. Moreover, the one-on-one setting helps pharmacists maintain focus during visits, minimizing external distractions.

When individuals respond to self-reported questionnaires, they encounter the possibility of misunderstanding both the questions and the available response choices. This, in turn, can potentially result in responses that lack validity.^50^ Contributors include factors like language barriers, ambiguity in question phrasing, and cognitive biases. Moreover, respondents may struggle to accurately recall relevant information or provide truthful responses due to social desirability bias. The use of the validated tools PSAD and DAVE in this research was therefore crucial.

In this research, pharmacists filled out the PSAD questionnaires immediately after the AD visits to ensure a high response rate and capture their fresh impressions. This approach, agreed upon with the pharmacy manager, resulted in a 94% response rate but may have introduced biases, as participants might not have had time to fully process the experience and could have been influenced by their immediate mood and sense of empowerment from the visit. The evaluations were kept anonymous to maintain reliability, with the detailer not having access to the PSAD-responses.

The items of the DAVE and PSAD questionnaires were in English, while the background questions and inquiries about the preferred method for knowledge updates were in Norwegian. As the DAVE and PSAD items are solely validated in English, the decision was made not to translate them into Norwegian. Given that most Norwegian pharmacists have a high level of proficiency in English, we believe that this choice does not introduce a significant bias.

In this study, for the question concerning the preferred method of knowledge update, respondents were asked to express their preferences on a scale from 1 (preference for AD) to 10 (preference for other methods). One limitation of this study is that the term “other methods” was not fully operationalized, leading to potential differences in interpretation among respondents. It would have been valuable to include a field in the questionnaire for respondents to specify their preferred alternative methods. Out of the 81 respondents, 4 individuals likely misunderstood the scale and selected values closer to 10 rather than closer to 1. This misinterpretation was inferred from the comments they provided in the open-ended text field of the questionnaire, where they expressed solely positive opinions about AD. However, as we could not definitively confirm this interpretation, their responses were not excluded, hence taking a conservative approach in the analysis about preferred knowledge update method.

The value of analyzing respondents’ comments in the open-ended text field lies in its capacity to reveal issues within the questionnaire that might create problems for respondents, even when they do not explicitly indicate misunderstandings.^7,51^ In the present study, the utilization of the open-ended text field contributed to identifying questions that could potentially affect data quality, in addition to provide support for the quantitative results.

The response rate of the PSAD questionnaire was 94%. However, despite the high participation rate, the results may not completely reflect respondents’ actual behavior, as research suggest a potential disparity between self-reported and observed behavior changes.^52,53^ In Smart et al,^33^ it is noteworthy that the research has not extensively explored the impact of the AD intervention on prescriber’s actual behavior. The collected data predominantly centers on prescribers’ self-reported assumed changes in behavior following the AD intervention. This limitation also applies to our study. Another limitation is the absence of an evaluation of patient experiences followed the AD intervention. For future research, it is recommended that AD interventions designed to induce behavior change also assesses the alignment between self-reported practice change and actual practice change. It would be valuable to explore the impact of AD on patients’ adherence to antibiotics. Previous studies have indicated changes in the quantity of prescribed drugs before and after such interventions.^7,29,30^ Nevertheless, there is a gap in research regarding how these changes affected patients’ adherence to the prescribed medications. Therefore, the impact of these changes on patients’ adherence remains unexplored.

The DAVE mean detailer scale summary score in this study was 12.45 (out of a maximum of 15). These findings clearly indicate that the detailer had a sense that the visits went smoothly, was content with own efforts, and perceived that the pharmacists responded positively to the key messages. While this high score is noteworthy, it’s important to note that DAVE scores are based on the detailer’s self-assessment of the performance during the AD visits. This self-assessment can present challenges when comparing scores between studies conducted in different countries, as individuals from diverse cultural backgrounds often rate themselves differently.^54,55^ Consequently, there may be variations in results when individuals from Norway and the United States, where the PSAD and DAVE instruments are developed, evaluate themselves and their performance using the same questionnaire. These variations can be attributed to cultural, social, and contextual factors that influence how individuals perceive and respond.^56^

In Smart et al,^33^ the in-person visits had an average duration of 19 min (SD 9). A systematic review that included 22 studies reported visit durations ranging from 16 to 31 min.^43^ In our study, the mean visit duration was 31 min, which is longer than the initially indicated 20 min during recruitment. However, the AD visits were designed to be a dialog between the pharmacist and the detailer. The detailer noted that when visits extended beyond the planned time, it was typically initiated by the pharmacists asking further questions or providing comments or reflections on the key messages. The time frame depended on the pharmacists’ interest and willingness to engage in the dialog. This experience was consistent with previous literature.^28^ As fostering engagement was desirable, a longer time frame for the visits was accepted.

An articulated concern regarding the AD updates was the perception that they required a substantial time commitment and were not well-aligned with pharmacy management practices. Managers encountered challenges in allocating approximately 30 min for one-on-one meetings with each employee during the workday. The primary reason for non-participation in the present AD campaign was the pharmacies’ lack of the necessary time and resources. There is a possibility that pharmacy managers and pharmacists who agreed to participate had a more favorable attitude toward AD compared to those who declined. Consequently, this could introduce a participant bias in the sample. However, it is important to note that the alternatives to AD as a knowledge update method, like group meetings, could be more time consuming in total for the pharmacy.^37^

A major strength of our study is that it was built upon a mixed embedded design, which offered new qualitative research on the topic^10,42^ to pharmacists through AD visits. The primary findings from the qualitative research were structured into 3 key messages, accompanied by descriptive illustrations, and presented in a brochure used as a framework for the AD dialog. Additionally, the brochure could be a valuable resource for pharmacists to reference after the AD visit. Another strength was that a single detailer carried out all 86 visits. This approach minimized potential biases arising from variations in detailer techniques, which could otherwise affect the results and the overall validity of the study.

## Conclusion

This study confirmed that AD is a successful knowledge updating tool for improving adherence promoting counseling among Norwegian pharmacists. Pharmacists appreciated one-on-one dialogs for tailored information, but the management cited time and resource constraints as barriers. AD emerged as the preferred method for knowledge updates among Norwegian pharmacists. Future research should investigate the alignment between self-reported intentions and actual practice behavior post-AD intervention, as well as its impact on patient outcomes.

## Supplemental Material

sj-docx-2-inq-10.1177_00469580241273228 – Supplemental material for Academic Detailing is a Preferred Knowledge Update Tool Among Norwegian Pharmacists to Improve Antibiotic Counseling: Results From a Quantitative Study Employing the Provider Satisfaction With Academic Detailing (PSAD) and the Detailer Assessment of Visit Effectiveness (DAVE) Tools Supplemental material, sj-docx-2-inq-10.1177_00469580241273228 for Academic Detailing is a Preferred Knowledge Update Tool Among Norwegian Pharmacists to Improve Antibiotic Counseling: Results From a Quantitative Study Employing the Provider Satisfaction With Academic Detailing (PSAD) and the Detailer Assessment of Visit Effectiveness (DAVE) Tools by Yngvild Kristine Rochette Bergsholm, Harald Christian Langaas, Tonje Krogstad and Lene Berge Holm in INQUIRY: The Journal of Health Care Organization, Provision, and Financing

sj-docx-3-inq-10.1177_00469580241273228 – Supplemental material for Academic Detailing is a Preferred Knowledge Update Tool Among Norwegian Pharmacists to Improve Antibiotic Counseling: Results From a Quantitative Study Employing the Provider Satisfaction With Academic Detailing (PSAD) and the Detailer Assessment of Visit Effectiveness (DAVE) Tools Supplemental material, sj-docx-3-inq-10.1177_00469580241273228 for Academic Detailing is a Preferred Knowledge Update Tool Among Norwegian Pharmacists to Improve Antibiotic Counseling: Results From a Quantitative Study Employing the Provider Satisfaction With Academic Detailing (PSAD) and the Detailer Assessment of Visit Effectiveness (DAVE) Tools by Yngvild Kristine Rochette Bergsholm, Harald Christian Langaas, Tonje Krogstad and Lene Berge Holm in INQUIRY: The Journal of Health Care Organization, Provision, and Financing

sj-docx-4-inq-10.1177_00469580241273228 – Supplemental material for Academic Detailing is a Preferred Knowledge Update Tool Among Norwegian Pharmacists to Improve Antibiotic Counseling: Results From a Quantitative Study Employing the Provider Satisfaction With Academic Detailing (PSAD) and the Detailer Assessment of Visit Effectiveness (DAVE) Tools Supplemental material, sj-docx-4-inq-10.1177_00469580241273228 for Academic Detailing is a Preferred Knowledge Update Tool Among Norwegian Pharmacists to Improve Antibiotic Counseling: Results From a Quantitative Study Employing the Provider Satisfaction With Academic Detailing (PSAD) and the Detailer Assessment of Visit Effectiveness (DAVE) Tools by Yngvild Kristine Rochette Bergsholm, Harald Christian Langaas, Tonje Krogstad and Lene Berge Holm in INQUIRY: The Journal of Health Care Organization, Provision, and Financing

sj-pdf-1-inq-10.1177_00469580241273228 – Supplemental material for Academic Detailing is a Preferred Knowledge Update Tool Among Norwegian Pharmacists to Improve Antibiotic Counseling: Results From a Quantitative Study Employing the Provider Satisfaction With Academic Detailing (PSAD) and the Detailer Assessment of Visit Effectiveness (DAVE) Tools Supplemental material, sj-pdf-1-inq-10.1177_00469580241273228 for Academic Detailing is a Preferred Knowledge Update Tool Among Norwegian Pharmacists to Improve Antibiotic Counseling: Results From a Quantitative Study Employing the Provider Satisfaction With Academic Detailing (PSAD) and the Detailer Assessment of Visit Effectiveness (DAVE) Tools by Yngvild Kristine Rochette Bergsholm, Harald Christian Langaas, Tonje Krogstad and Lene Berge Holm in INQUIRY: The Journal of Health Care Organization, Provision, and Financing
