# Supplementary material for: Academic Detailing is a Preferred Knowledge Update Tool Among Norwegian Pharmacists to Improve Antibiotic Counseling: Results From a Quantitative Study Employing the Provider Satisfaction With Academic Detailing (PSAD) and the Detailer Assessment of Visit Effectiveness (DAVE) Tools
Source: Inquiry. 2024 Sep 4;61:00469580241273228. doi: 10.1177/00469580241273228 (PMC11375677; doi:10.1177/00469580241273228)
Supplement: sj-docx-2-inq-10.1177_00469580241273228 – Supplemental material for Academic Detailing is a Preferred Knowledge Update Tool Among Norwegian Pharmacists to Improve Antibiotic Counseling: Results From a Quantitative Study Employing the Provider Satisfaction With Academic Detailing (PSAD) and the Detai [file sj-docx-2-inq-10.1177_00469580241273228.docx]

**Supplementary file DAVE questionnaire**

**Detailer Perception of the Quality of the Interaction**

After your academic detailing session, please mark a box indicating your response for each of the questions below.

|  | Not at all | Slightly | Moderately | Very | Extremely |
| --- | --- | --- | --- | --- | --- |
| 1. The visit was useful to the provider | □ | □ | □ | □ | □ |
| 2. The provider is willing to implement the key points | □ | □ | □ | □ | □ |
| 3. The provider is likely to change his/her/their practice as a result of this visit | □ | □ | □ | □ | □ |
| 4. It is feasible for the provider to implement the key points | □ | □ | □ | □ | □ |
| 5. The conversation went smoothly | □ | □ | □ | □ | □ |
