# Supplementary material for: Academic Detailing is a Preferred Knowledge Update Tool Among Norwegian Pharmacists to Improve Antibiotic Counseling: Results From a Quantitative Study Employing the Provider Satisfaction With Academic Detailing (PSAD) and the Detailer Assessment of Visit Effectiveness (DAVE) Tools
Source: Inquiry. 2024 Sep 4;61:00469580241273228. doi: 10.1177/00469580241273228 (PMC11375677; doi:10.1177/00469580241273228)
Supplement: sj-docx-3-inq-10.1177_00469580241273228 – Supplemental material for Academic Detailing is a Preferred Knowledge Update Tool Among Norwegian Pharmacists to Improve Antibiotic Counseling: Results From a Quantitative Study Employing the Provider Satisfaction With Academic Detailing (PSAD) and the Detai [file sj-docx-3-inq-10.1177_00469580241273228.docx]

**Supplementary file PSAD questionnaire**

Questionnaire used for pharmacists after the AD visit. Parts of the questionnaire was in Norwegian, but has been translated to English in this supplementary file.

**Academic Detailing in community pharmacies**

To you who have completed an Academic Detailing visit. Thank you for participating. We now ask that you complete this questionnaire. Your answers are completely anonymous and cannot be traced back to you. It takes approximately two to five minutes to complete the questionnaire.

An Academic Detailing (AD) visit is a scientifically documented method for knowledge updating. The assumption is that one-on-one training provides higher learning outcomes than other training methods. In Norway, RELIS has previously conducted Academic Detailing visits for general practitioners. In this research study, we aim to determine if the method is also suitable for community pharmacists. It is therefore important for our research that you answer this questionnaire as honestly as possible.

The questionnaire consists of three introductory questions, ten questions about your experience of the Academic Detailing visit, and a general question about this method of knowledge updating. Finally, you have the opportunity to leave comments if you wish. The second part of the questionnaire is validated and developed in the USA and is therefore in English.

**Part 1**

Gender:

.

Pharmaceutical education:

Master’s degree

Bachelor’s degree

Other

*If “Other” was ticked, this question appears:*

You have indicated “Other” under Pharmaceutical education. Please specify here:

.

Years of practicing as a community pharmacist:

.

**Part 2**

Please mark the box indicating your response below for each of the following questions after your academic detailing session.

|  | Not at all | Slightly | Moderately | Very | Extremely |
| --- | --- | --- | --- | --- | --- |
| 1. The detailer was knowledgeable | □ | □ | □ | □ | □ |
| 2. The detailer was an effective communicator | □ | □ | □ | □ | □ |
| 3. Academic detailing is an effective way to get updated on important topic(s) | □ | □ | □ | □ | □ |
| 4. The printed/electronic detailing material was useful | □ | □ | □ | □ | □ |
| 5. I would be receptive to future visits | □ | □ | □ | □ | □ |
| 6. This topic was relevant to my practice | □ | □ | □ | □ | □ |
| 7. This is an important topic | □ | □ | □ | □ | □ |
| 8. The key messages are feasible to implement in my practice | □ | □ | □ | □ | □ |
| 9. The key messages were consistent with my practice | □ | □ | □ | □ | □ |
| 10. My practice is likely to change as a result of this visit | □ | □ | □ | □ | □ |

**Part 3**

**Preferred method for knowledge updating in your daily work**

Consider the method of knowledge updating you have just experienced and compare it with other methods you normally use to update your knowledge in your daily work. Indicate on a scale from 1 to 10 which method you prefer, where 1 indicates that you most prefer Academic Detailing visits, and 10 indicates that you most prefer other methods that you usually use.

| 1  Academic Detailing  □ | 2  □ | 3  □ | 4  □ | 5  □ | 6  □ | 7  □ | 8  □ | 9  □ | 10  Other methods  □ |
| --- | --- | --- | --- | --- | --- | --- | --- | --- | --- |

If you have any comments you would like to share with us, you can do so here

L .
