# Supplementary material for: Academic Detailing is a Preferred Knowledge Update Tool Among Norwegian Pharmacists to Improve Antibiotic Counseling: Results From a Quantitative Study Employing the Provider Satisfaction With Academic Detailing (PSAD) and the Detailer Assessment of Visit Effectiveness (DAVE) Tools
Source: Inquiry. 2024 Sep 4;61:00469580241273228. doi: 10.1177/00469580241273228 (PMC11375677; doi:10.1177/00469580241273228)
Supplement: sj-docx-4-inq-10.1177_00469580241273228 – Supplemental material for Academic Detailing is a Preferred Knowledge Update Tool Among Norwegian Pharmacists to Improve Antibiotic Counseling: Results From a Quantitative Study Employing the Provider Satisfaction With Academic Detailing (PSAD) and the Detai [file sj-docx-4-inq-10.1177_00469580241273228.docx]

**Model of a Typical Academic Detailing Visit as performed in this research**

1. **Introduction and Purpose:**
   - The visit began with the detailer introducing herself, emphasizing her affiliation with xxx (censored due to anonymity), and explaining the purpose of the visit. She clearly outlined the expected duration of the meeting and ensured that the location was available for the entire session.
   - She spent a few minutes creating a relaxed and friendly atmosphere to put the pharmacist at ease. She emphasized that this was not a test or evaluation but an opportunity for a dialogue about antibiotic dispensing in primary healthcare and to present the latest research in the field.
2. **Overview of Research and Materials:**
   - The detailer explained that she had conducted a qualitative study involving focus group interviews with general practitioners, pharmacists, and patients. She presented the main findings using the brochure, holding it up as she described it. (It is important not handing out the brochure until the end of the visit to maintain the pharmacist’s focus during the dialogue).
   - She mentioned that the leaflet in the brochure contained useful general information about antibiotics.
3. **Initiating Dialogue:**
   - The conversation began with the detailer presenting the key messages and main themes from the brochure. She then asked: "Are there any of these topics you would like to discuss in more detail or spend more time on?"
   - This approach allowed the conversation to be guided by the pharmacist’s interests and needs. When there were no specific preferences, she proceeded from the beginning of the brochure.
4. **Engaging Discussion:**
   - Engagement was often sparked by the figures in the brochure, particularly the scenarios in the doctor's office where patients strive to be seen, heard, and understood. Discussing the initial closed question approach *"Have you used this antibiotic before, yes/no?"* and transitioning to the open question *"What information did your doctor give you about this antibiotic?"* generated significant interest. This approach raised awareness of their role and opportunities to optimize the dialogue with the patient, thereby ensuring correct antibiotic use.
   - The detailer highlighted how these questions could enhance patient-centred care and triangulated dialogue by starting the consultation with an open-ended question. Pharmacists appreciated having practical tools to improve communication tailored to patient needs, beginning with the doctor’s consultation.
5. **Tailoring Information:**
   - Throughout the visit, the detailer ensured the information was tailored to the individual pharmacist’s needs through continuous two-way communication. She observed body language and ensured active dialogue. When the pharmacist became passively listening, she asked activating follow-up questions such as: *"What is your experience with this area?"*, *“Which situation do you see as most common when dispensing antibiotics?”* or *"How do you handle such situations?"*
   - This method ensured the conversation was tailored to the pharmacist’s needs. The brochure guided the visit, but the depth and focus on specific points depended on the pharmacist's preferences.
6. **Conclusion:**
   - In the end of the dialogue, key messages were repeated. The detailer thanked the pharmacist for their time and provided them with the physical brochure. She asked if they had any questions about the discussion and encouraged them to reach out with any follow-up questions, showing her contact information on the brochure.
   - She asked if there was anything from the discussion they planned to implement in their practice, and then provided a QR code for the PSAD survey and encouraged them to fill it out as soon as possible, acknowledging the busy nature of pharmacy work.
