# Supplementary material for: Academic Detailing is a Preferred Knowledge Update Tool Among Norwegian Pharmacists to Improve Antibiotic Counseling: Results From a Quantitative Study Employing the Provider Satisfaction With Academic Detailing (PSAD) and the Detailer Assessment of Visit Effectiveness (DAVE) Tools
Source: Inquiry. 2024 Sep 4;61:00469580241273228. doi: 10.1177/00469580241273228 (PMC11375677; doi:10.1177/00469580241273228)
Supplement: sj-pdf-1-inq-10.1177_00469580241273228 – Supplemental material for Academic Detailing is a Preferred Knowledge Update Tool Among Norwegian Pharmacists to Improve Antibiotic Counseling: Results From a Quantitative Study Employing the Provider Satisfaction With Academic Detailing (PSAD) and the Detail [file sj-pdf-1-inq-10.1177_00469580241273228.pdf]

1 Sources of Knowledge on Antibiotic Use and Antimicrobial Resistance

Patients' Main Sources of Knowledge:

- Digital information
- Written information
- Face-to-face meetings

Common Misconceptions:

- Antibiotics are started without a doctor's consultation
- Individuals, not bacteria, become resistant
- Leftover antibiotics are thrown in the trash or flushed down the toilet
- Importance of time intervals for the effectiveness of antibiotics

Pharmacy pharmacists need to be aware of these misconceptions in their counseling.

2 The Patient's Experience of a Doctor's Visit for an Infectious Disease

- Reduced general condition
- Main focus is to present their history and explain their symptoms in the hope of a quick recovery
- Feeling of vulnerability during the consultation makes it difficult to receive information
- The doctor does not routinely ensure that the information is understood
- Time pressure/busyness

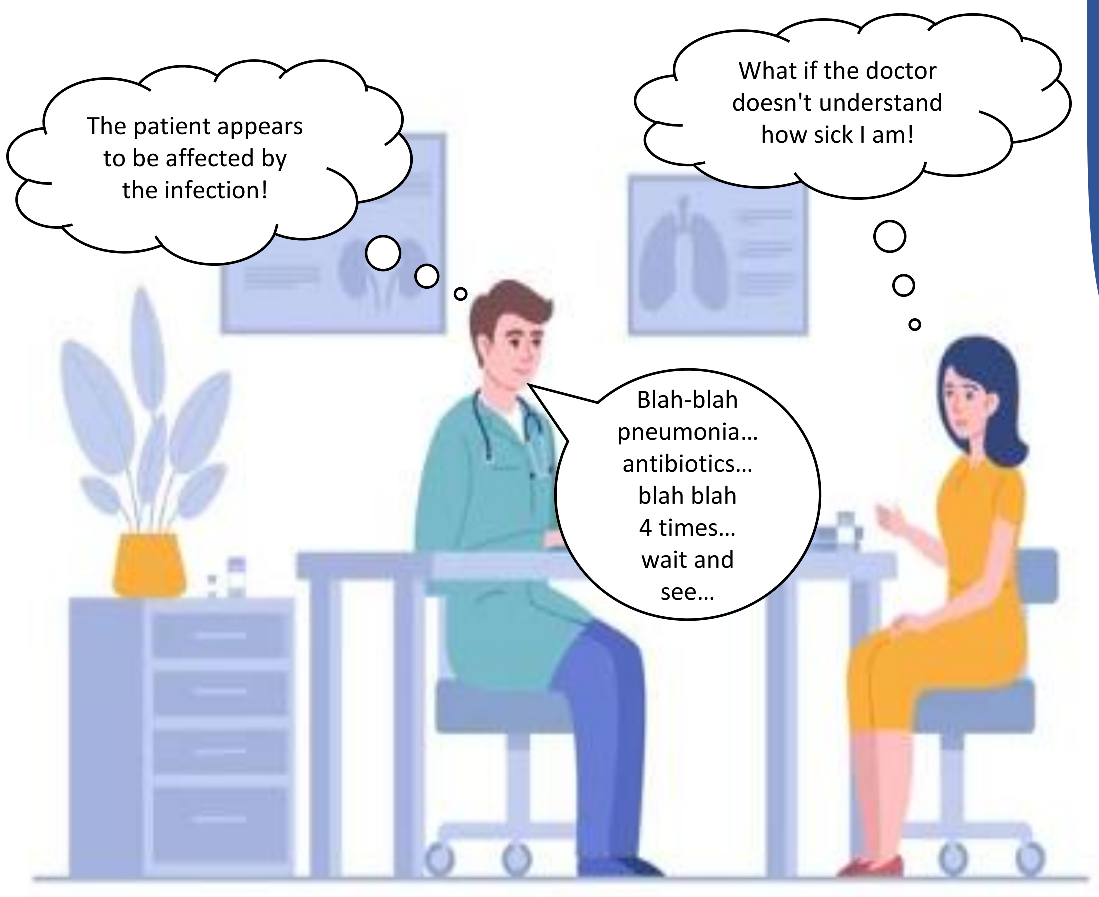

Figure 1: At the GP's office (macrovector/Freepik.com)

3 The patient's request to the pharmacist

The patient feels empowered after their symptoms have been acknowledged by the doctor. They are receptive to information at the pharmacy when picking up the antibiotics.

- Positive that the pharmacist uses simple language
- Tailor information to individual needs, avoid autopilot information
- Start the patient meeting with an open question: What information have you received from the doctor? instead of closed questions, such as: Have you used this before?
- Leverage the opportunity when the patient is receptive to information

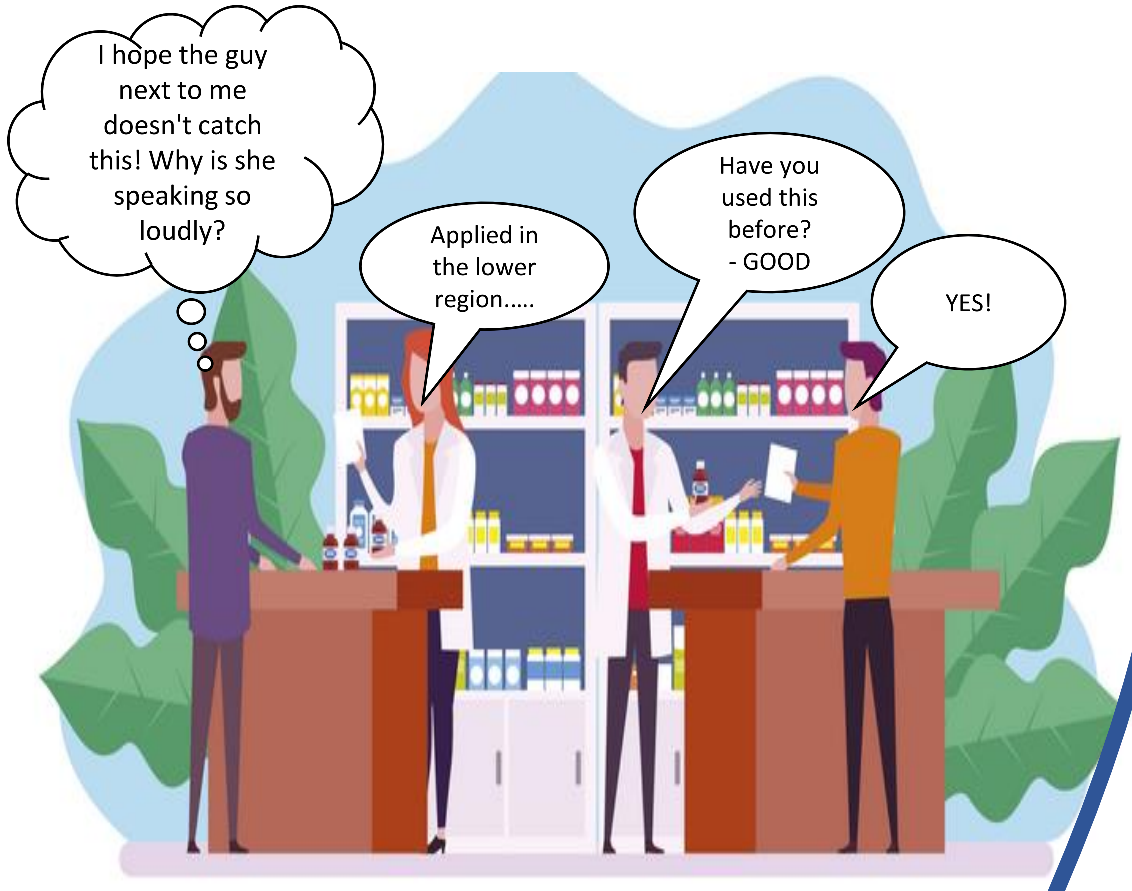

Figure 2: At the pharmacy (macrovector/Freepik.com)

4 Barriers to patient-centered communication to ensure proper antibiotic use

At the doctor's office:

- Time, the consultation becomes too stressful
- Feeling of vulnerability makes it difficult to receive the information provided. Thoughts are elsewhere
- Difficult medical terms and expressions

At the pharmacy:

- Lack of information such as indication and "wait and see" on the prescription.
- Extensive use of closed-ended questions.
- Should utilize the pharmacy's consultation room for conducting sensitive conversations

5 Information the patient desires from the pharmacist

The patient seeks information about the prescribed antibiotic

- Dosage
  - Timing during the day
- Dosage interval
  - Time between doses (time-dependent vs. concentration-dependent bactericidal effect)
- Precautions
  - Food, dairy products, sun exposure, alcohol, etc...
- Expected effect
  - When to expect the effect to occur
  - What to do if the effect is absent
- Common side effects
  - Nausea, diarrhea, and fungal infections
  - What measures can be taken?
- Residual antibiotics and environmental impact
  - Information on how to dispose of surplus antibiotics
- What is resistance
  - Antibiotics should only be used when indicated/needed, and only then (4) The mantra of completing the course seems outdated, as "surplus antibiotics" seem to drive resistance (5)
  - It is the bacteria that become resistant, not individuals
- Multiple courses?
  - If the patient has had multiple treatments, patients want to know what differentiates the newly prescribed from the previous one

Understanding increases motivation to adhere to the treatment

The content of the brochure is based on research studies conducted by researchers at censored (1,2) and current guidelines for antibiotic use in primary healthcare (3).

The brochure was crafted by the following individuals:

- Censored
- Cencored
- Cencored
- Cencored

March 2023

References

1. Cencored due to anonymity
2. Cencored due to anonymity
3. The Norwegian Directorate of Health. National clinical guidelines for antibiotic use in primary healthcare http://www.helsebiblioteket.no/retningslinjer/antibiotika/ (Published: November 2012).
4. The Department of eHealth. Antibiotic and antimicrobial resitance: HelseNorge; 2022 [updated 17.01; cited 2022 16.11]. Available from: https://www.helsenorge.no/medisiner/antibiotika-og-resistens/om-antibiotikaresistens/
5. Spellberg B. The New Antibiotic Mantra-"Shorter Is Better". JAMA Intern Med. 2016 Sep 1;176(9):1254-5. doi: 10.1001/jamainternmed.2016.3646. PMID: 27455385; PMCID: PMC5233409
6. Aanonsen S. og Heim, J. [What are the causes of poor adherence to antibiotics in Norway, and will improving the information given to patients at pharmacies enhance their adherence to treatment]? Masters' thesis, Department of Pharmacy, The Faculty of Health Sciences, Copenhagen University, 2022
7. European Centre for Disease Prevention and Control. http://ecdc.europa.eu/en/Pages/home.aspx
8. NORM/NORM-VET 2021: Usage of Antimicrobial Agents and Occurrence of Antimicrobial Resistance in Norway. Norwegian Institute of Public Health and Norwegian Veterinary Institute; 2022.
9. Costelloe C, Metcalfe C et al. Effect of antibiotic prescribing in primary care on antimicrobial resistance in individual patients: systematic review and meta-analysis. BMJ 2010; 340: c2096.
10. Langaas HC, Eriksen AK. [Correct use of penicillin]. Nor Farmaceut Tidsskr 2014; 122(3): 26 -7
11. Nordeng, H. og Spigset, O. [Pharmaceuticals and the correct use]. Gyldendal, Oslo, 2007
12. Drug Allergy: Diagnosis and Management of Drug Allergy in Adults, Children and Young People. National Clinical Guideline Centre (UK). London: National Institute for Health and Care Excellence (UK); 2014 Sep

Questions? Contact us here:

Sensored due to anonymity

6 Patients are requesting a triangular dialogue

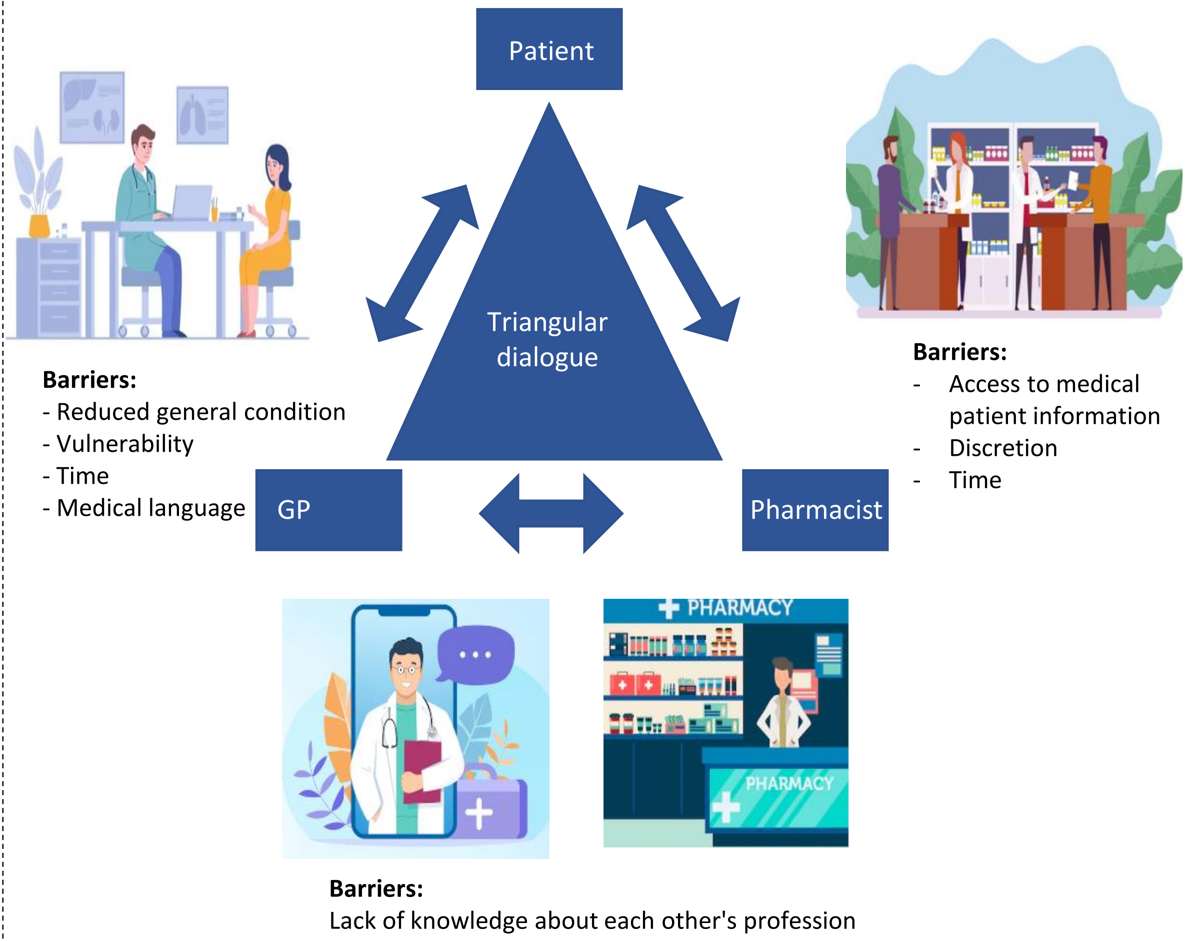

Figure 3: Triangular dialogue (macrovector/Freepik.com)

Recommendations for the pharmacist:

- Assess <u>body language</u>. How receptive is this patient to information? Balance what information must be provided and what information can be provided.
- Start the patient meeting with an open question: '<u>What information have you received from the doctor?</u>' and based on the patient's response: confirm, supplement, inform, or correct.
- This approach makes it easier to adhere to the doctor's prescription. Work in collaboration with the doctor.
- There is a lot of learning for the patient in <u>recounting</u> what they remember from the information given at the doctor's office. Allow the patient the opportunity to do so, be patient.
- Acknowledging, for example, not remembering any information given by the GP often leads to the patient paying extra attention when the pharmacist provides information.
- A good dialogue between pharmacist and patient can ensure that the <u>information is tailored</u> to the individual's need for information so that the antibiotic is used more correctly

The five most common main reasons for poor adherence to antibiotic treatment (6)

1. Forgetting to take it
2. Feeling better
3. Fear of side effects
4. Fear of the development of antibiotic resistance
5. Difficulty in taking antibiotics at specific times

AD
Academic Detailing

Logo censored

Dispensing of antibiotics at pharmacies

MAIN MESSAGE:

- Tailored patient information from the pharmacy can contribute to more appropriate use of antibioticsi
- Initiate the conversation with: *"What information have you received from the doctor about this antibiotic"*?.
  ➡ Confirm, supplement, inform, and correct.
- Patients are receptive, motivated, and empowered in their belief in rapid recovery when picking up antibiotics.

The content of the brochure is based on research studies conducted by researchers at (censored) (1,2) and current guidelines for antibiotic use in primary healthcare (3).

March 2023

ATTACHMENT TO AD-BROCHURE
Academic detailing

Logo censored

Dispensing of antibiotics at pharmacies

Antibiotic resistance

All modern medicine relies on effective antibiotics. A bacterial strain is resistant when it can tolerate higher concentrations of antibiotics than can be achieved at the site of infection. This poses a significant risk of treatment failure

The European Centre for Disease Prevention and Control (ECDC) estimates that approximately 25,000 people die each year in Europe due to antibiotic-resistant microorganisms (7). In Norway, we use less antibiotics than many of our European neighbors

85% of total antibiotics are used in primary healthcare (3, 4, 8)

NOTE! Ensure that you dispense the correct number of tablets/capsules or quantity of suspension to the patient. Also, choose the smallest package that contains enough tablets/capsules/suspension. If there are too many tablets in the package, the patient must be informed about the return of antibiotics.

Antibiotic targets

Penicillin inhibits cell wall synthesis by targeting peptidoglycan synthesis. Peptidoglycan constitutes 90% of the cell wall in gram-positive bacteria such as pneumococci and streptococci. These are the most common bacteria in respiratory infections.

The Mycoplasma and Chlamydia families do not have cell walls, so we use macrolides or tetracyclines to inhibit ribosomes from synthesizing proteins. The bacteria cannot reproduce as a result.

Time-dependent bacterial killing.
For some types of antibiotics, bacterial killing depends on the time the antibiotic concentration exceeds the bacteria's minimum inhibitory concentration (MIC). This applies to beta-lactams (e.g., penicillins), tetracyclines, and macrolides. Good clinical and microbiological effects can be expected if the concentration exceeds the MIC for more than 50% of the time. Antibiotics with time-dependent bacterial killing should be dosed frequently, while it is less important for each dose to be high.

Did you know that: After an antibiotic course, there is an increased risk of colonization and infection with bacteria resistant to the antibiotic used for up to 12 months after completing the course? This, in turn, leads to increased use of broad-spectrum antibiotics in the next infection. The more courses, the longer the resistance persists.

Antibiotic targets, cont.

Concentration-dependent bacterial killing
means that bacterial killing increases with increasing concentration. This is particularly true for aminoglycosides and fluoroquinolones (ciprofloxacin), but such agents are rarely used in general practice

When should one take penicillin?
Phenoxymethylpenicillin taken orally has a very short half-life (approximately 30-60 minutes) and should therefore be dosed at least 4 times a day. Note: consistent dosing intervals. Penicillin can be taken with or without meals

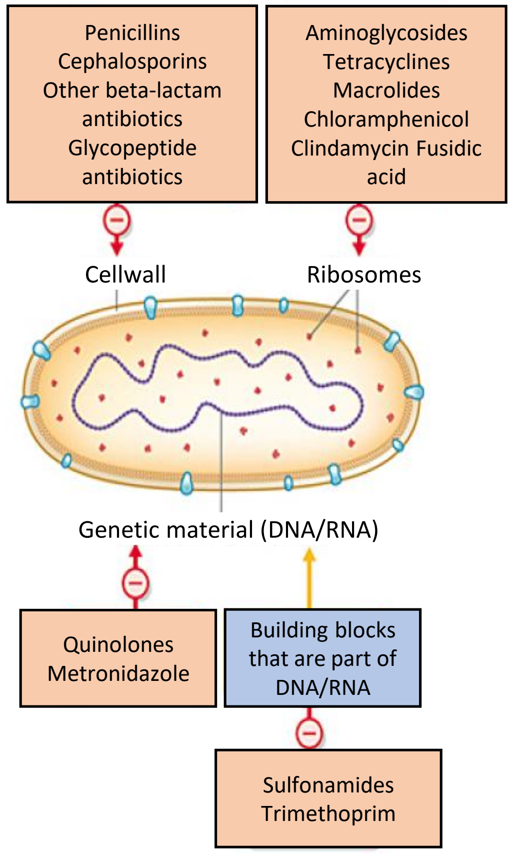

Figure 4 : Antibiotic targets (11)

Penicillin for children

Many parents find it difficult to give penicillin to children due to its very bitter taste. This should generally not be used as an argument for prescribing broader-spectrum antibiotics.

Tips for administering penicillin to small children:
- Inform parents that penicillin suspension tastes unpleasant and that it's important for them to motivate the children.
- Suspensions or drops should not be mixed with food or drink. It's better to offer food or drink that the child likes (e.g., juice, yogurt, ice cream) immediately after penicillin administration both to remove the unpleasant taste and as a reward.
- Drops provide less volume and a more intense unpleasant taste, but they can often be easier than suspension, which requires a much larger volume.
- It's advantageous to administer suspensions and drops with a measuring syringe far back in the throat.
- Ask parents if they think the child can swallow tablets. The smallest strength of penicillin tablets is small and can be given from the age of 5. Penicillin tablets should preferably be swallowed whole, as this will also mask the taste. The tablet can be hidden in a spoonful of jam or yogurt, but should not be crushed or chewed

Penicillin allergy

Penicillin allergy exists and can be serious in some cases, but in most cases, it's only mild, nonspecific, and transient symptoms. When asked about allergy, the doctor should inquire thoroughly to determine if it could be an immediate allergic reaction. In such cases, penicillins should be avoided. If there are no indications of an immediate allergic reaction, there is no reason to emphasize undocumented information about allergy. Around 10% of patients report having a penicillin allergy, but of these, only 1 in 10 have a genuine allergy, so the remaining 9 will tolerate penicillin.

Side effects

Advice for managing reactions during ongoing penicillin treatment:
- **Rash without itching**: Treatment can continue. The patient is informed to notify if the rash worsens. Penicillin can be used later without allergological evaluation.
- **Rash with itching or mild urticaria:** Discontinue the antibiotic and consider whether there is an indication for further treatment. Evaluation is necessary before penicillin can be given again. (Consultation with a doctor)
- **Severe urticaria or urticaria with swelling in the face and joints, blisters and sores on mucous membranes, or widespread severe skin reaction**: Discontinue treatment, administer antihistamines and possibly steroids (refer to a doctor). These patients should never receive penicillin again
